# A temperature shock can lead to trans-generational immune priming in the Red Flour Beetle, *Tribolium castaneum*

**DOI:** 10.1002/ece3.1443

**Published:** 2015-02-27

**Authors:** Hendrik Eggert, Maike F Diddens-de Buhr, Joachim Kurtz

**Affiliations:** Institute for Evolution and Biodiversity, Westfälische Wilhelms-Universität MünsterHüfferstraße 1, Münster, DE-48149, Germany

**Keywords:** *Bacillus thuringiensis*, heat shock, insect immunity, stress, trans-generational effects, *Tribolium castaneum*

## Abstract

Trans-generational immune priming (TGIP) describes the transfer of immune stimulation to the next generation. As stress and immunity are closely connected, we here address the question whether trans-generational effects on immunity and resistance can also be elicited by a nonpathogen stress treatment of parents. General stressors have been shown to induce immunity to pathogens within individuals. However, to our knowledge, it is as of yet unknown whether stress can also induce trans-generational effects on immunity and resistance. We exposed a parental generation (mothers, fathers, or both parents) of the red flour beetle *Tribolium castaneum*, a species where TGIP has been previously been demonstrated, to either a brief heat or cold shock and examined offspring survival after bacterial infection with the entomopathogen *Bacillus thuringiensis*. We also studied phenoloxidase activity, a key enzyme of the insect innate immune system that has previously been demonstrated to be up-regulated upon TGIP. We quantified parental fecundity and offspring developmental time to evaluate whether trans-generational priming might have costs. Offspring resistance was found to be significantly increased when both parents received a cold shock. Offspring phenoloxidase activity was also higher when mothers or both parents were cold-shocked. By contrast, parental heat shock reduced offspring phenoloxidase activity. Moreover, parental cold or heat shock delayed offspring development. In sum, we conclude that trans-generational priming for resistance could not only be elicited by pathogens or pathogen-derived components, but also by more general cues that are indicative of a stressful environment. The interaction between stress responses and the immune system might play an important role also for trans-generational effects.

## Introduction

Parasites with their negative effects on host fitness are a strong evolutionary force (Schulenburg et al. 2009). Therefore, hosts have evolved a wide repertoire of defences, which are often adjusted to prior pathogenic experience (Schmid-Hempel 2005; Siva-Jothy et al. 2005; Rolff 2007). These defences can also protect offspring, which are often exposed to the same parasites as their parents (Agrawal et al. 1999). Such trans-generational defences are demonstrated in vertebrates (Harvell 1990; Mousseau and Fox 1998; Agrawal et al. 1999; Grindstaff et al. 2003; Rolff 2007) and invertebrates, where they are denoted as trans-generational immune priming (Little and Kraaijeveld 2004; Sadd et al. 2005; Moret 2006; Sadd and Schmid-Hempel 2007, 2009; Tidbury et al. 2011; Zanchi et al. 2011). Such trans-generational effects are mostly transmitted by mothers (Harvell 1990; Mousseau and Fox 1998; Agrawal et al. 1999; Grindstaff et al. 2003), but in some cases, also fathers provide protection (Reid et al. 2006). In insects, these defences can come with specificity for the type of pathogen encountered (Roth et al. 2010), but often also involve unspecific, broad-spectrum immune traits (Moret 2006; Roth et al. 2010; Zanchi et al. 2011; Moreau et al. 2012). It is therefore relevant to know to what extent such trans-generational responses are stressor-specific or also elicited by general indicators of nonpathogen stress, rather than specific immunological insults. Overall, any factor causing a possibly negative impact on an organism can be classified as stress. This includes not only the exposure to pathogens (Gillespie et al. 1997; Hoffmann 2003; Rolff and Reynolds 2009) but also temperature (Lee and Denlinger 1991; Chown and Nicolson 2004; Gullan and Cranston 2009; Denlinger and Lee 2010; Tattersall 2012), dehydration (Danks 2000), or low-quality diet (Siva-Jothy and Thompson 2002; Srygley et al. 2009; Myers et al. 2011). Mechanistically, the stress response is signaled through membrane damage, misfolded proteins, or DNA damage, which induce stress-activated protein kinase pathways (Stronach and Perrimon 1999; Tibbles and Woodgett 1999; Inoue et al. 2001; Wang et al. 2003). The stress and the immune response in turn are tightly connected in insects, such that immune insults lead to elevated expression of stress proteins (Altincicek et al. 2008; Adamo 2010; Freitak et al. 2012) and *vice versa* (Adamo 2008; Zhang et al. 2011; Marshall and Sinclair 2012). In *Galleria mellonella,* it is shown that mild physical and/or thermal stress leads to short-term immune priming, protecting the larvae against infection with *Aspergillus fumigatus*. Furthermore, treated *G*. *mellonella* larvae show an increase in haemocyte density and elevated expression of prophenoloxidase and two antimicrobial peptides (Browne et al. 2014). However, little is known about the influence of stress on the immune system in the context of trans-generational effects. Triggs and Knell found that diet has strong parental effects on offspring immunity (Triggs and Knell 2012). The effect of a brief temperature shock (to elicit the stress response) on offspring immunity has, to our knowledge, not been studied before.

We here investigated the effect of a short temperature stress on offspring immunity and resistance. Importantly, we aimed to discriminate between maternal and paternal effects. We exposed *T*. *castaneum* males or females to a cold or heat shock and observed their offspring's survival after bacterial challenge with *Bacillus thuringiensis* (Bt), as well as phenoloxidase activity (PO). PO is an important enzyme in insect immunity and is correlated with resistance to pathogens in a variety of species (Stanley and Kim 2011). Moreover, we investigated costs by measuring parental fecundity and an important offspring fitness component, developmental time.

## Material and Methods

### The model system

Due to its small size and short generation time, the model system *T. castaneum* is very suitable to investigate ecology, behavior, and immunology of host–parasite interactions. As a primarily grain dwelling organism and therefore major pest of stored cereals, *Tribolium spp*. are found worldwide (Fedina and Lewis 2008). Juveniles and adults aggregate in big groups and share the same environment. Adults are long-lived and females lay eggs continuously over their life (Sokoloff 1974). Naturally, *T. castaneum* harbors a range of protozoans and other parasites (West 1960; Blaser and Schmid-Hempel 2005; Fischer and Schmid-Hempel 2005).

For the experiment, we used the Croatia 1 (Cro1) beetle line. Cro1 was collected in 2010 in Croatia (Milutinovic et al. 2013) and since then kept under standard conditions in our laboratory (30°C, 70% humidity, and a 12-h/12-h light/dark cycle).

We use *Bacillus thuringiensis* (Bt), (strain DSM No. 2046) as a microparasite, which was obtained from the German Collection of Microorganisms and Cell Cultures (DSMZ). Bt is an insect-specific pathogen, which was isolated from the Indian meal moth, *Plodia interpunctella*, and is known to affect the fitness of *T. castaneum* (Abdel-Razek et al. 1999; Milutinovic et al. 2013).

### The temperature shock experiment

For the temperature shock experiments, eggs of *T. castaneum* were individually distributed into 96-well plates, filled with flour and 5% yeast. Animals were raised at 30°C, 70% humidity, and a 12-h/12-h light/dark cycle. Individuals were checked regularly for their developmental stage. When the pupal stage was reached, animals were sexed and distributed individually into fresh 96-well plates. Six weeks after the distribution of eggs, all individuals had reached sexual maturity.

Two separate experiments for heat shock and cold shock were performed under otherwise identical conditions. Male and female beetles were randomly assigned to a temperature shock treatment or left naïve 24 h before mating. In the heat shock experiment, beetles were kept for 1 h at 40°C. For the cold shock, beetles were kept for 2 h at 4°C. The following treatment groups with respect to sex were formed in each experiment: (1) both sexes naïve (none); (2) female naïve and male temperature shock (paternal); and (3) female temperature shock and male naïve (maternal); and (4) female and male temperature shock (both). The breeding pairs (*n* = 40 per treatment group) were allowed to oviposit for 4 days, sieving off the offspring every other day. Offspring were kept individually distributed in 96-well plates.

### Developmental time

To check for differences in developmental time, five individuals per pair were checked daily until they reached the adult stage. Day of pupation and day of eclosion were monitored.

### Survival after bacterial challenge

Offspring survival of bacterial infection (in days postchallenge) was measured as a phenotypic outcome of the effect of the parental stress. One adult individual per pair and of each treatment group was randomly assigned to one of the three challenge treatments (Naïve, PBS, Bt). Challenge was performed with live bacteria, which were grown as described in Roth & Kurtz (Roth et al. 2009) and adjusted to a cell concentration of 10^11^ per mL in PBS solution. Animals in the sterile PBS group were treated similarly, except that the PBS solution contained no bacteria. In total, we challenged 480 beetles (40 pairs × 4 treatment groups × 3 challenge treatments) per experiment. Thereafter, beetles were randomly and individually distributed to 96-well plates filled with flour and 5% yeast. After the challenge, survival of the animals was checked daily for 3 days and on days 5 and 7.

### Phenoloxidase assay

Further, the activity of phenoloxidase (*V*_max_), a key enzyme in insect immunity, was measured to directly determine immune system activity. Phenoloxidase as part of the humoral immune responses to infection, invasion, and wounding is responsible for the melanization reaction (Stanley and Kim 2011). Three adult individuals per pair were pooled into one sample. The hemolymph was collected by puncturing the pleural membrane between pronotum and occiput with a sterile hypodermic needle. The out-flowing droplet of hemolymph was collected in a sterile, prechilled glass capillary. Pools of hemolymph were handled as described in Roth & Kurtz (Roth et al. 2009). Briefly, for each pooled sample, we collected 0.1 *μ*L of hemolymph, flushed it into a well of a 96-well plate containing 20 *μ*L Bis–Tris buffer (0.1 mol/L, pH 7.5), and stored at −80°C. To determine phenoloxidase (PO) activity, 50 *μ*L of *aqua dest* and 50 *μ*L Bis–Tris buffer were given into wells of a 96-well plate (flat bottom) with 20 *μ*L of the hemolymph in Bis–Tris buffer, prepared as described above. After adding 50 *μ*L of L-Dopa (4 mg/mL L-Dopa dissolved in Bis–Tri buffer), absorbance was measured on a Tecan Infinite M200 plate reader (Tecan Group Ltd., Männedorf, CH) at 490 nm at 37°C for 90 min, once every minute. Phenoloxidase activity was determined as the fastest change in absorbance over 15 min. (*V*_max_).

### Fecundity assay

Fecundity was measured for an extra subset of seven pairs per treatment as the total number of viable offspring after an oviposition period of 21 days.

### Statistics

All statistical analyses were performed in JMP 10 (SAS Institute Inc. Cary, NC, USA). Statistics were performed separately for each experiment.

To test for differences of survival rates between treatment groups, only the dataset of Bt-challenged animals in the F1 generation was used. In the PBS and naïve challenge group, only three of 320 animals died. For survival, a generalized linear model was applied with treatment (naive, paternal, maternal, both) as fixed factor. The response variable was the state of the animals (dead, alive) at the end of the experiment (day 7) using a binomial error distribution. A post hoc contrast test was used to test the parental temperature shock treatment groups (maternal, paternal, and both) against the control group (none).

Phenoloxidase measurements were Box–Cox transformed to achieve normal distribution. Differences in phenoloxidase activity were thereafter analyzed in an ANOVA with treatment group as fixed factor. Tukey's HSD post hoc test was used subsequently (Table1).

**Table 1:** Post hoc tests for significant main effects of (a) survival, (b) phenoloxidase activity (PO), and (c) development

(a) Survival			
Cold shock			
Contrast			
Treatment [none]	1	1	1
Relatedness priming [maternal]	−1	0	0
Relatedness priming [paternal]	0	−1	0
Relatedness priming [both]	0	0	−1
Chi-square test	1.93	3.36	10.87
*P*	0.165	0.067	<0.001

(b) PO		
Cold shock		
Tukey's HSD post hoc test	Least square mean	Different
None	−0.445	B
Maternal	−0.396	A
Paternal	−0.417	A B
Both	−0.396	A

Heat shock		
Tukey's HSD post hoc test	Least square mean	Different
None	−0.296	A
Maternal	−0.377	B
Paternal	−0.366	B
Both	−0.386	B

(c) Development				
Cold shock	Pupae		Adult	
Post hoc test Steel's method	*Z*	*P*	*Z*	*P*
Maternal	6.68	<0.001	2.37	0.0475
Paternal	5.99	<0.001	1.55	0.2810
Both	−7.42	<0.001	−4.39	<0.001

Heat shock	Pupae		Adult	
Tukey's HSD post hoc test	Least square mean	Different	Least square mean	Different
None	20.735	C	28.245	B
Maternal	22.36	B	29.02	A
Paternal	21.86	B	28.435	A B
Both	23.1	A	29.025	A

To test for differences in developmental time in experiment 1, an ANOVA was performed with mean of either day of pupation or day of eclosion per pair as response variable and treatment group as factor. Tukey's HSD post hoc test was used subsequently. As the data were not normally distributed, a Kruskal–Wallis test was performed to test for differences in developmental time in experiment 2. The response variable was day of pupation or day of eclosion per pair (median). In post hoc test, we compared the control group against the treatment groups using Steel's method.

The total number of offspring of each female was monitored by counting all offspring. To test for differences in fecundity, an ANOVA was carried out with treatment as factor and fecundity as response variable.

## Results

### Survival upon bacterial challenge

#### Cold shock

**Figure 1:**
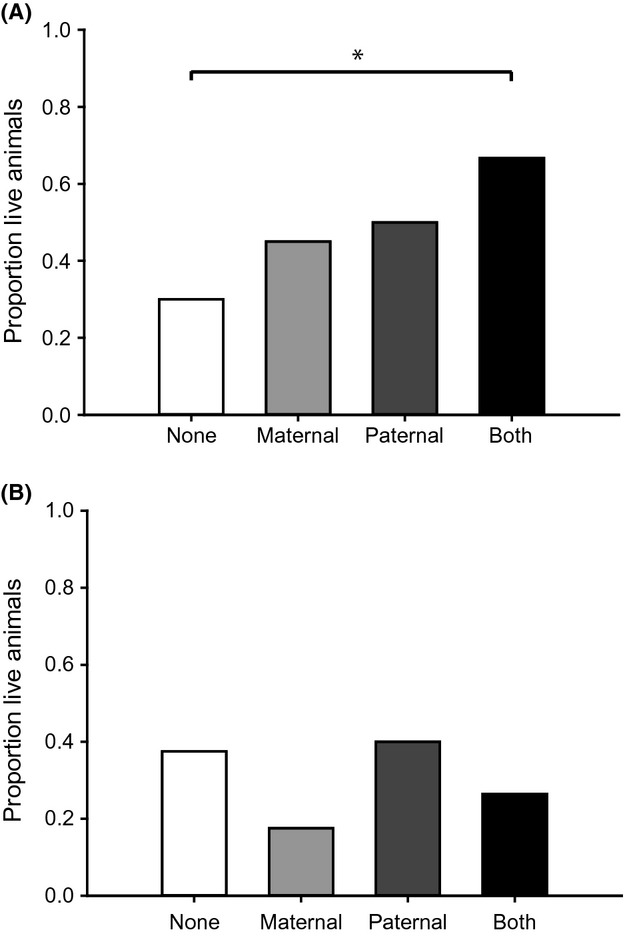
Result of a bacterial challenge in *Tribolium castaneum* offspring after parental exposure to either cold (A) or heat (B) shock. One offspring of each pair per treatment was either randomly assigned to bacterial challenge (*n* = 40), to sham treatment with PBS (*n* = 40), or left naïve as control (*n* = 40). Asterisks show significantly different survival rates between the treatments.

Bacterial challenge of offspring with *B. thuringiensis* led to average survival rates between 30 and 67% for the different treatments in the cold shock experiment (Fig.1A). The data showed a significant effect of the parental treatment on offspring survival (GLM, chi-square test = 11.092; df = 3; *p*_3_ = 0.0112). When both parents had been exposed to cold shock, survival of offspring was highest compared to offspring survival of naïve parents (GLM, chi-square test = 10.886; df = 3; contrast: *p*_3_ = 0.0009).

#### Heat shock

Bacterial challenge of offspring with *B. thuringiensis* led to average survival rates between 17 and 40% for the different treatments in the heat shock experiment (Fig.1B). To analyze whether parental experience of temperature stress has an effect on the offspring immune system, we categorized the offspring according to the parental temperature treatment (both naïve, paternal, maternal, both heat shock). There was no significant difference of offspring survival between the different treatment groups, but offspring with mothers exposed to heat shock show a tendency for the lowest survival rate (GLM, chi-square test = 6.319; df = 3; *p*_3_ = 0.0971).

### Phenoloxidase activity (PO)

#### Cold shock

**Figure 2:**
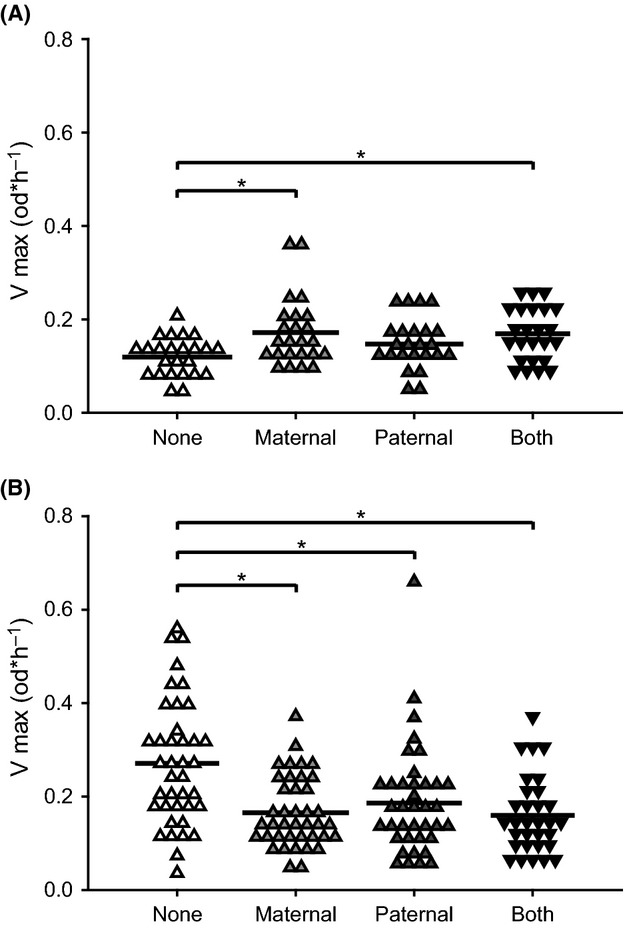
Constitutive PO measurement of hemolymph samples taken from naïve offspring. Either the parental generation received a cold (A) or heat (B) shock. Heat shock/cold shock: none (*n* = 39/24); maternal (*n* = 40/24); paternal (*n* = 38/24); and both (*n* = 38/24) Asterisks show significantly different PO between the treatments.

Parental cold shock exposure affected the PO of offspring significantly (ANOVA, *F* Ratio = 4.35; df = 3; *p*_3_ = 0.0065). PO was increased when the mother or both parents were exposed to cold shock, as compared to naïve parents (Fig.2A).

#### Heat shock

The experience of heat shock in the parental generation had an effect on the constitutive phenoloxidase activity (PO) in offspring (Fig.2B). PO was significantly decreased when any of the parents were exposed to heat shock, as compared to naïve parents (ANOVA, *F* Ratio = 7.567; df = 3; *p*_3_ = 0.0001).

### Developmental time

#### Cold shock

**Figure 3:**
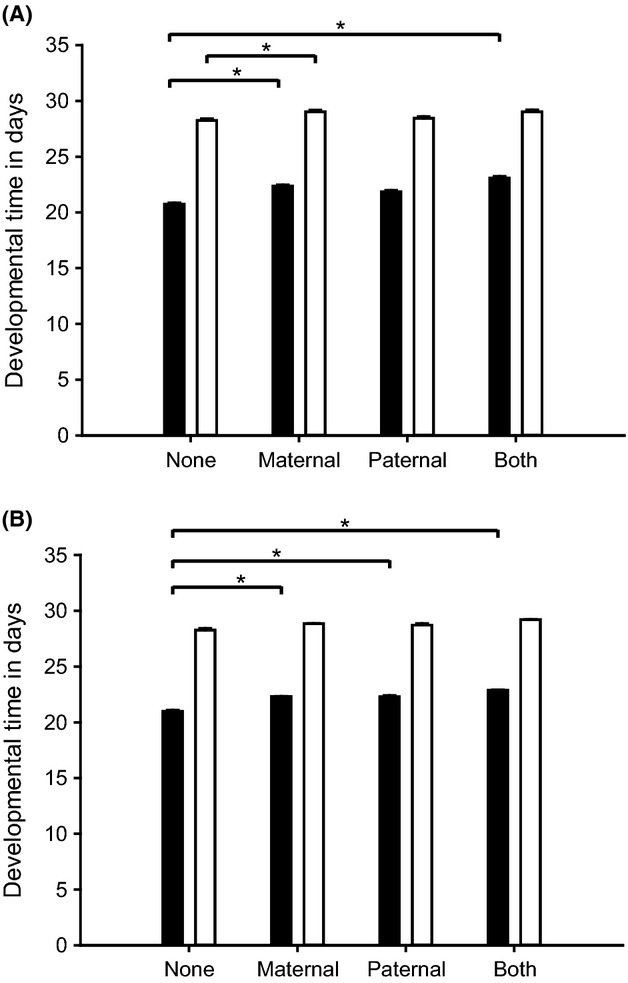
Developmental time of offspring, which parents were exposed to cold (A) or heat (B) shock. Twelve individuals of five pairs per treatment (*n* = 60) were checked daily until they reached the adult stage. Black bars show time in days until pupation; white bars show the developmental time until adult stage. Asterisks show significantly different PO between the treatments.

Also, the experience of a cold shock in the parental generation significantly affected the developmental time of the next generation. Until pupation, offspring of cold shock-treated parents needed 2 days longer on average (Kruskal–Wallis test, chi-square test = 97.204; df = 3; *p*_3_ = 0.0001). Developmental time of naïve offspring until eclosion to the adult stage was 1 day faster (Kruskal–Wallis test, chi-square test = 43.974; df = 3; *p*_3_ = 0.0001) than that of offspring of cold-exposed parents (Fig.3A).

#### Heat shock

The developmental time of offspring sired by parents who were exposed to heat shock was significantly prolonged (ANOVA, *F* Ratio = 50.951; df = 3; *p*_3_ = 0.0001). The time was even longer when both parents were heat-shocked (Fig.3B). The time until eclosion was also significantly affected by parental heat shock, when the mother or both parents were exposed to heat shock (ANOVA, *F* Ratio = 5.556; df = 3; *p*_3_ = 0.0012).

### Total number of offspring

#### Cold shock

The parental exposure to cold shock had no significant effect on the total number of offspring (ANOVA, *F* Ratio = 2.137; df = 3; *p*_3_ = 0.136).

#### Heat shock

The total number of offspring was not significantly affected by the parental exposure to heat shock (ANOVA, *F* Ratio = 0.228; df = 3; *p*_3_ = 0.876).

## Discussion

Trans-generational immune priming provides offspring facing a persistent infection risk with a beneficial immune activation across generations. But it is likely to be a plastic trait dependent on the host or pathogen life history and the specific host–pathogen interaction (Little and Kraaijeveld 2004; Tidbury et al. 2011). In some insects, females transfer immunity (Sadd et al. 2005; Moreau et al. 2012); in others, also males are able to induce protection to the next generation (Roth et al. 2010; Zanchi et al. 2011); and yet some insect species do not show the effect at all (Voordouw et al. 2008; Linder and Promislow 2009). In addition, it seems that not only the way of transfer but also the level of specificity between maternally and paternally derived immune protection is different (Roth et al. 2010). On the other hand, also general indicators of stress such as diet affect offspring immunity (Triggs and Knell 2012). The underlying mechanisms are rarely understood, and we know very little about other stressors that may affect trans-generational immune priming.

In our study, we tested if a short temperature shock as an unspecific cue for stress can mediate the phenomenon of trans-generational immune priming. We hereby show for the first time that a short temperature shock has trans-generational effects on the immune system of the next generation. Cold exposure in the parental generation of *T. castaneum* leads to increased survival after bacterial infection in the offspring. Our data support the hypothesis of a cross talk between the response to cold and immune stress, as both trigger trans-generational effects resulting in better survival of the offspring after bacterial infection. However, the effect of cold shock on offspring immunity observed in the present study cannot explain the specificity for bacterial species that was observed for maternal trans-generational immune priming in the same host (Roth et al. 2010). Freitak et al. (2014) recently suggested that maternal trans-generational immune priming might be mediated by the direct deposition of bacteria into the eggs. This, however, may not explain priming by other agents than bacteria, nor can it explain paternal immune priming. We thus hypothesize that the different forms of trans-generational priming might be based on diverse mechanisms (Eggert et al. 2014).

Studies of *Drosophila melanogaster* show that flies exposed to short cold shock survive better when infected with either bacteria (Linder et al. 2008) or fungi (Le Bourg et al. 2009), compared to flies that were not exposed. Furthermore, transcriptomes of cold-shocked flies and heat-shocked beetles reveal the up-regulation of immune genes also in the absence of pathogens (Altincicek et al. 2008; Zhang et al. 2011; Freitak et al. 2012; Marshall and Sinclair 2012). Noteworthy, this was looked at within one generation and not across generations.

In line with the enhanced survival of offspring of cold-exposed beetles is the constitutive phenoloxidase (PO) activity in our experiment. As a general but fast innate immune component, we found that offspring of cold-shocked parents, especially mothers, show an elevated PO. Yet the same PO-driven process that helps to defend against infectious agents within the hemocoel also induces melanin synthesis and cuticle darkening (Cerenius and Söderhäll 2004). This physiological link is referred to as the “temperature-dependent immune investment hypothesis,” which predicts that the need for darker cuticles in the cold (to increase absorption of solar radiation) comes with an elevated PO-based immune defense (Fedorka et al. 2013).

We propose that trans-generational immune priming may share common mechanisms with the stress response. Similar to a more general priming by fathers (Roth et al. 2010), nonpathogen stressors such as cold shock induce survival benefits after bacterial infection and elevated constitutive PO in offspring. This is also the case in paternally derived transfer of immune priming in *Plodia interpunctella* where nutritional stress via parental diet shows strong trans-generational effects on PO. In contrast to the effect of cold shock, we cannot find a significant effect of heat shock on offspring survival and all offspring of heat-exposed parents show a decreased PO, which could also be a direct consequence of parental heat shock on offspring physiology rather than an immunity-mediated effect. However, empirical evidence suggests that higher temperatures do increase a variety of immune responses (Ouedraogo et al. 2003; Freitak et al. 2012) and can induce physiological trade-offs within one generation, in other species–pathogen interactions (Bensadia et al. 2006; Adamo and Lovett 2011).

It is likely that stress-induced immune activation comes at a cost in the absence of infection and that mounting a costly immune response may affect both generations. Stressed parents may face a resource allocation trade-off as the transfer of substances to the egg or sperm is energetically costly (Grindstaff et al. 2003). In our study, we consider costs of parental temperature shock for the parents by measuring fecundity, and for the offspring by recording their developmental time. Neither cold nor heat shock affected the fecundity of the parental generation. But if the parents, and more specifically the mothers, are exposed to cold or heat shock, the offspring show prolonged development. This can be a direct effect of the parental treatment or an allocation trade-off between offspring development and immunity. However, the costs of paternal temperature shock are comparably low in contrast compared to the maternal costs.

In sum, we show that trans-generational priming for resistance might be induced by cues that are indicative of nonpathogen stress. This suggests that connections between stress responses and immunity that were previously identified within generations are also relevant across generations. Fitness benefits might be associated with a costly up-regulation of immunity in situations where general stressors are indicative of prospectively harmful situations and or of high pathogen exposure.
